# Bilateral Thigh Myonecrosis in Diabetes: A Case Report

**DOI:** 10.7759/cureus.89600

**Published:** 2025-08-08

**Authors:** Mohammed A Alkubaisi, Jeyanthy Rajkanna

**Affiliations:** 1 Medicine, Peterborough City Hospital, Peterborough, GBR; 2 Diabetes and Endocrinology, Peterborough City Hospital, Peterborough, GBR

**Keywords:** clinical dermatology, diabetic patient, endocrine disorders, general radiology, myositis

## Abstract

Diabetic myonecrosis is an uncommon complication of poorly controlled diabetes mellitus. Usually, patients present with acute painful swelling of the affected muscles, mostly the quadriceps muscle of the thigh. We present the case of a 57-year-old male with type 2 diabetes mellitus who presented with progressive bilateral thigh pain and reduced movement. Blood results indicated slightly elevated creatine kinase, C-reactive protein, and erythrocyte sedimentation rate levels, and poor diabetes control. Magnetic resonance imaging showed features suggestive of myonecrosis. A muscle biopsy demonstrated active myofiber necrosis consistent with diabetic myonecrosis. The patient’s symptoms improved after 11 days of treatment with intravenous fluids, painkillers, glycemic control, and physical therapy. This will highlight the importance of suspecting diabetic myonecrosis in patients presenting with bilateral acute swollen leg pain with a background of uncontrolled diabetes mellitus.

## Introduction

Diabetic mellitus affects 5.6 million people in the UK, with 90% having type 2 diabetes, 8% having type 1 diabetes, and 2% having other rare types [1]. Diabetic myonecrosis is an uncommon complication of uncontrolled diabetes mellitus [2]. Angervall and Stener first described diabetic myonecrosis in 1965 [3]. Patients present with non-specific symptoms such as acute onset of pain, tenderness, and swelling of the affected muscles [4]. Diabetic myonecrosis affects diabetic patients who may also have microvascular complications, including retinopathy and nephropathy [5]. Clinical examination, blood tests, and radiological findings may be enough for the diagnosis of diabetic myonecrosis [6]. Muscle biopsy is needed when there is no improvement with the conservative treatment [5]. Treatment is usually supportive, and the disease is self-limiting, but recurrence can occur in around 50% [4]. The short-term prognosis is good, but the long-term prognosis is poor, with most patients dying within five years. [2]. We report the case of a patient who presented with bilateral thigh diabetic myonecrosis nine years after being diagnosed with type 2 diabetes mellitus.

## Case presentation

A 57-year-old male presented to the emergency department with bilateral proximal lower limb weakness, swelling, and pain, which started five to six weeks before admission to the hospital. This affected his mobility and movement, especially when changing from sitting to a standing position. There was no weakness, sensory loss, or swelling of the upper limbs. He did not report skin rash, flu-like symptoms, or recent trauma. He is known to have uncontrolled hypertension, uncontrolled type 2 diabetic mellitus since 2015, diabetic retinopathy, and hypercholesterolemia. His medications were amlodipine 10 mg once daily, candesartan 32 mg once daily, metformin 500 mg twice daily, and atorvastatin 20 mg once daily. He was a non-smoker and drank alcohol occasionally.

**Figure 1:**
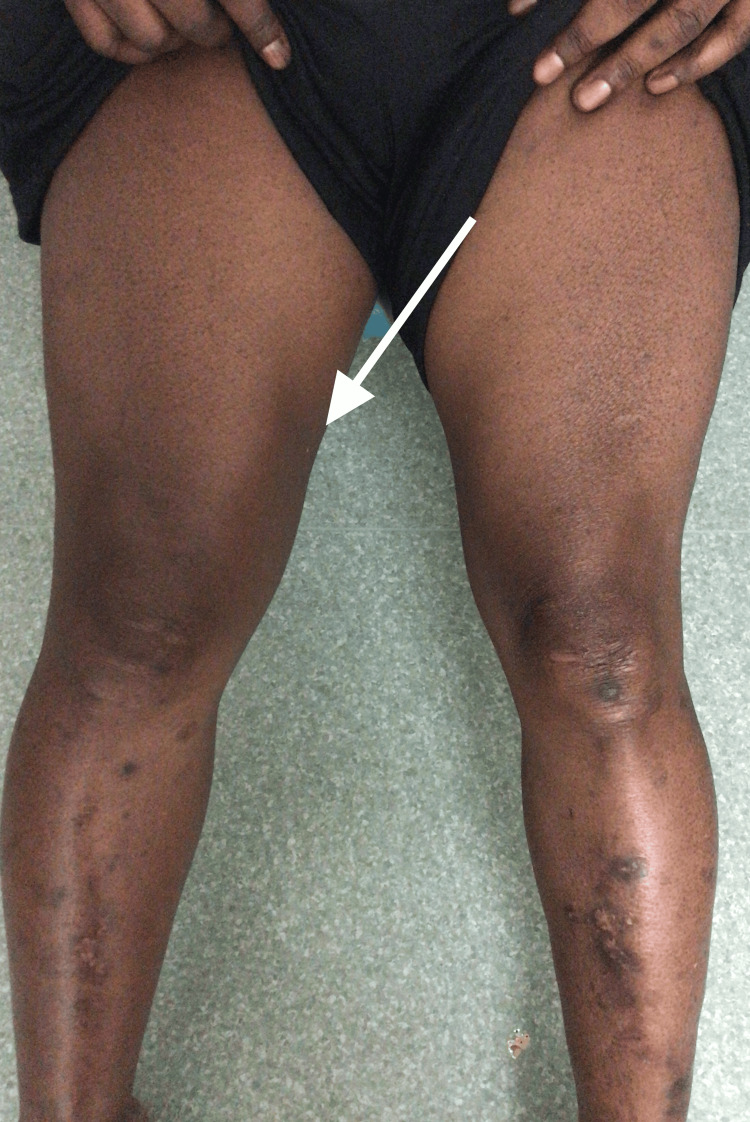
Picture of lower limbs showing bilateral swelling of both thighs with more on the right (white arrow), as well as diabetic dermopathy.

On examination, his blood pressure was 178/116 mmHg, his heart rate was 98 beats per minute, respiratory rate was 16 per minute, oxygen saturation was 98% on air, and his temperature was 36.8 degrees. Lower limbs examination showed bilateral swelling and tenderness of the lower thighs and around the knees, with more on the right medial thigh (Figure 1). There was evidence of proximal myopathy (could not stand from a sitting position without support) with power 3/5 and diminished knee reflexes bilaterally. There was no sensory loss. Rest of the neurological examination, including upper limbs, was unremarkable.

Investigations

**Figure 2:**
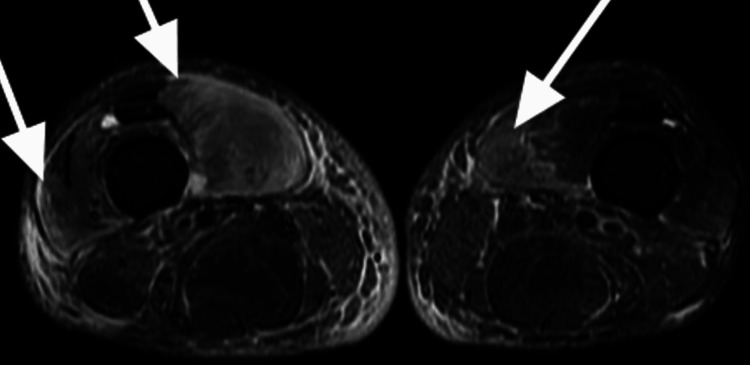
T2-weighted cross-sectional magnetic resonance imaging (MRI) of both thighs showing diffuse edema in the vastus medialis and vastus lateralis muscles on the right thigh, with edema noted in the vastus medialis muscle on the left thigh (white arrows).

**Figure 3:**
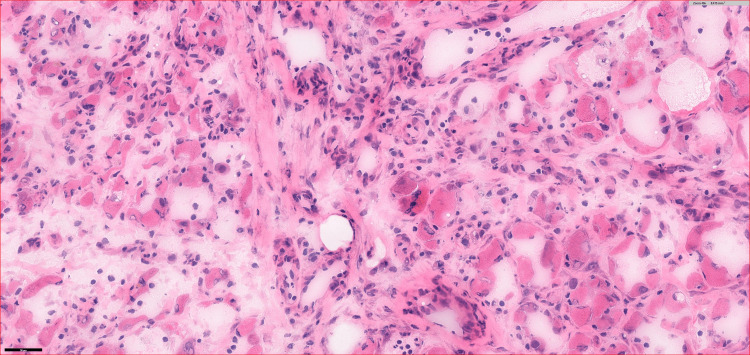
Histology (H&E stain) of the right calf showing diffused skeletal muscle myonecrosis, with surrounding edema, fibrosis, and inflammatory cell infiltration‌.

Blood results indicated slightly elevated creatinine kinase, C-reactive protein, and erythrocyte sedimentation rate levels, and poor diabetes control (Table 1). Given his presentation with bilateral leg swelling and uncontrolled diabetic mellitus, a magnetic resonance imaging of both legs was performed (Figure 2). A biopsy of the right thigh showed severe inflammation, necrosis, and fibrosis (Figure 3).

**Table 1:** Blood test results

Blood test	Result	Normal ranges
White blood cell	6.2	4.5-11.1×10^9^/L
Hemoglobin	135	130-180 g/L
Glycated hemoglobin	100	<41 mmol/mol
Creatinine kinase	418	40-320 U/L
C-reactive protein	10	<5 mg/L
Creatinine	70	59-104 umol/L
Erythrocyte sedimentation rate	60	0-15 mm/hr
Urine albumin: creatinine ratio	212	<30 mg/mmol creatinine
Antibodies including OJ antibodies, Ro52 antibodies, MI-2B antibodies, MI-2a antibodies, JO-1 antibodies, antinuclear antibodies, anti-neutrophil cytoplasmic antibodies, anti-muscle kinase antibody, HMG reductase antibodies	Negative	-

Treatment

The patient was started on doxazosin due to uncontrolled hypertension, and the dose was increased to 4 mg once daily. His glycated hemoglobin (HbA1c) on admission was 100 mmol/mol, and his previous HbA1c from 2017 to 2022 was in the range of 53 to 95 mmol/mol. The plan was to start him on insulin for his poor glycemic control; however, the patient declined it. Then he was started on gliclazide 160 mg twice daily and dapagliflozin 10 mg once daily. He was treated with paracetamol 1 gm four times daily and codeine phosphate 30-60 mg when needed to control the leg pain. Atorvastatin was stopped due to suspected 3-hydroxy-3-methyl coenzyme A (HMG-CoA) reductase myopathy.

The patient felt clinically better, and his swelling and tenderness were getting better. He was discharged after 11 days and scheduled for outpatient follow-up with the rheumatology, nephrology, and physiotherapy clinics.

Outcome and follow-up

**Figure 4:**
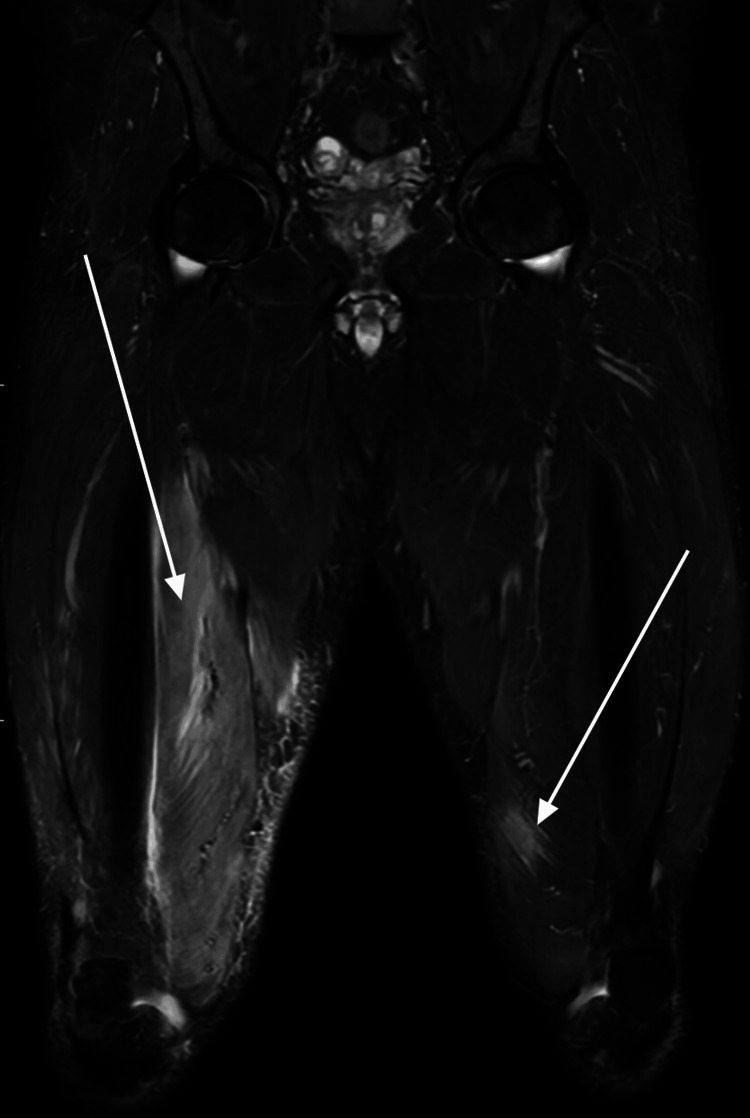
T2-weighted coronal MRI of both thighs showing improvement of left thigh muscle edema and worsening of right thigh muscle edema (white arrows).

MRI scan of lower limbs was repeated after three months, which showed the right vastus medialis muscle edema to be more extensive and more intense than on the previous examination. There was a minor improvement in the left-sided muscle changes (Figure 4). His Hba1c improved to 48 mmol/mol after three months with the new diabetic medications.

## Discussion

Diabetic myonecrosis can be seen in both diabetic mellitus types 1 and 2 and occurs bilaterally in 10% of cases [7]. The average age at which symptoms begin typically falls between 42 and 45 years, and the duration from diabetes diagnosis to the appearance of diabetic myonecrosis spans from 15 to 20 years. However, in our case, it happened after nine years and in both legs. Diabetic myonecrosis predominantly develops in individuals with poorly managed diabetes, who have a recorded HbA1c level greater than 9% (75 mmol/mol) at the time of diagnosis [8]. Diabetic myonecrosis mostly affects the lower limbs, with quadriceps in 60%, hip adductors in 15%, and hamstrings in 10% [4]. Although the exact pathogenesis of diabetic myonecrosis is unknown, it is thought to include ischemia-reperfusion injury, vasculitis alterations, hypercoagulability, and vasculopathy changes brought by poorly managed long-term diabetes. It is believed that endothelium injury initiates the process, causing tissue ischemia, which sets off an inflammatory chain reaction that culminates in ischemic necrosis [9].

The blood investigations generally show high erythrocyte sedimentation rate, normal white blood cell count, and normal or mild elevation of creatine phosphokinase, but these are non-specific [5]. Magnetic resonance imaging findings are invariably characterized by increased signal intensity of the diffusely enlarged muscle groups on T2-weighted sequences and gadolinium-enhanced images [5].

The differential diagnosis of such a presentation will be cellulitis, auto-immune conditions such as polymyositis and dermatomyositis, statin-induced myonecrosis (as our patient was on statin), spinal cord compression, and trauma.

The patient was afebrile, and his white blood cells and C-reactive protein were normal, unlikely to be cellulitis. His creatinine kinase was normal, with normal muscle antibodies and no skin rash or upper limb involvement, thus unlikely to be polymyositis or dermatomyositis. For his lower limb weakness and hyporeflexia, he underwent spinal magnetic resonance imaging, which ruled out spinal cord compression or radiculopathy. Statin-induced myonecrosis was excluded as the anti-HMG-CoA reductase antibody was negative.

As mentioned previously, magnetic resonance imaging of both legs showed bilateral swelling of the vastus medialis, and the thigh muscle biopsy showed necrosis. Both these findings are supportive of the diagnosis of diabetic myonecrosis.

## Conclusions

Diabetic myonecrosis is underdiagnosed due to its rarity. Clinicians should suspect it in patients who have uncontrolled, longstanding diabetes present with swollen and acute pain in the limbs. MRI is the investigation of choice to detect the increased signal intensity in the affected muscles. There is evidence that the results presented here may help physicians to diagnose muscle necrosis without the need for invasive procedures and that imaging should be the preferred diagnostic method because of the risks associated with open muscle biopsy.
